# Polypoid Lesion Segmentation Using YOLO-V8 Network in Wireless Video Capsule Endoscopy Images

**DOI:** 10.3390/diagnostics14050474

**Published:** 2024-02-22

**Authors:** Ali Sahafi, Anastasios Koulaouzidis, Mehrshad Lalinia

**Affiliations:** 1 Department of Mechanical and Electrical Engineering, Digital and High-Frequency Electronics Section, University of Southern Denmark, 5230 Odense, Denmark; melal@sdu.dk; 2 Surgical Research Unit, Odense University Hospital, 5000 Svendborg, Denmark; anastasios.koulaouzidis@rsyd.dk; 3 Department of Clinical Research, University of Southern Denmark, 5230 Odense, Denmark; 4 Department of Medicine, OUH Svendborg Sygehus, 5700 Svendborg, Denmark; 5 Department of Social Medicine and Public Health, Pomeranian Medical University, 70204 Szczecin, Poland

**Keywords:** polypoid lesion identification, polypoid lesion segmentation, YOLO-V8, WCE images, gastrointestinal disorders, colorectal cancer, artificial intelligence

## Abstract

Gastrointestinal (GI) tract disorders are a significant public health issue. They are becoming more common and can cause serious health problems and high healthcare costs. Small bowel tumours (SBTs) and colorectal cancer (CRC) are both becoming more prevalent, especially among younger adults. Early detection and removal of polyps (precursors of malignancy) is essential for prevention. Wireless Capsule Endoscopy (WCE) is a procedure that utilises swallowable camera devices that capture images of the GI tract. Because WCE generates a large number of images, automated polyp segmentation is crucial. This paper reviews computer-aided approaches to polyp detection using WCE imagery and evaluates them using a dataset of labelled anomalies and findings. The study focuses on YOLO-V8, an improved deep learning model, for polyp segmentation and finds that it performs better than existing methods, achieving high precision and recall. The present study underscores the potential of automated detection systems in improving GI polyp identification.

## 1. Introduction

**Figure 1:**
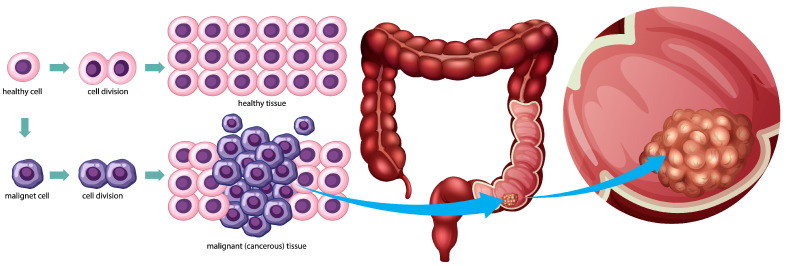
Progression of CRC [6].

Gastrointestinal (GI) tract disorders present a significant public healthcare issue in Europe, resulting in around 1 million deaths annually; aside from considerable mortality, GI pathology imposes a substantial burden on healthcare macroeconomics [1]. With a global ageing trend, it is expected that the impact of these conditions will steadily increase in the future [2]. Typically, GI tract cancers initiate as small, benign growths known as polyps, which have the potential to progress into cancer over time. Small-bowel tumours (SBTs) are relatively rare, representing around 5% of GI tract neoplasms with a variable incidence [3]. Although the prevalence varies [4], the non-specific, subtle clinical presentation of SBTs often makes their diagnosis challenging. Timely identification of SBTs is crucial for improving prognosis, considering that a conclusive diagnosis often involves employing a combination of diagnostic methods, such as wireless capsule endoscopy (WCE). Conversely, Colorectal Cancer (CRC) stands out as a widespread yet preventable form of cancer. The prognosis of cancer patients hinges on a multitude of factors, exhibiting variability contingent upon the stage and site of the malignancy, usually ranging from 48.6% to 59.4% [5]. As stated previously, the majority of CRC cases develop slowly from polyps, specifically adenomatous polyps, as depicted in Figure 1. The prompt removal of these polyps can efficiently prevent the advancement of cancer and decrease cancer-related mortality by as much as 70%. However, there is a considerable range in the adenoma detection rate (varying from 7% to 53%) owing to differences in situational awareness, technical skill, and detection aptitude among endoscopists.

WCE is a relatively new development in GI endoscopy. The modality involves utilising a swallowable miniature imaging device to capture digital images of the GI tract [7]. These images are transmitted to a portable recording device, downloaded with the assistance of relevant software, and subsequently scrutinised by gastroenterologists for pathology. A typical WCE session generates tens of thousands of images based on a working battery life of 8–12 h. Therefore, an effective and precise automated identification method would alleviate clinicians from the onerous task of analysing a large volume of images per patient. Computer-aided technologies, notably computer vision, offer a means of automating the analysis of WCE videos, leading to reduced processing time and examination efforts for physicians. The present research has primarily concentrated on addressing two key challenges pertinent to the analysis of GI endoscopic images: the identification and differentiation of malignant tissue. The former pertains to the identification of malignant abnormalities within the intestinal tract, encompassing conditions such as tumours, polyps, ulcers, and other manifestations primarily bleeding.

The present investigation represents an important leap forward in the domain of polypoid segmentation within WCE images. Employing artificial intelligence, particularly leveraging the innovative YOLO-V8 methodology, this study addresses the critical demand for heightened precision and efficiency in detecting polypoid lesions. It introduces a valuable clinical tool with the objective of minimising overlooked diagnoses, facilitating early identification, and contributing significantly to cancer prevention. The evaluation of the YOLO-V8 spans its diverse iterations, denoted as YOLO-V8 n, s, m, l, and x. Our results, revealing exceptional precision, recall, and mean average precision, underscore the effectiveness of this approach compared to other cutting-edge deep learning-based models. Beyond its current influence, this study establishes the foundation for forthcoming investigations into identifying and segmenting polypoid lesions. In the swiftly changing realm of computer vision, creating a benchmark dataset is increasingly recognised as a crucial requirement.

The subsequent sections of this paper are structured as follows: Section 2 explores the related works, providing an overview of relevant studies. Section 3 presents the materials used and the applied methodology. Experimental results and discussions are elaborated upon in Section 4. Finally, Section 5 serves as the conclusion, summarising the key findings and insights of the paper.

## 2. Related Studies

In the subsequent section, we embark on a comprehensive examination of prevailing methodologies for the analysis of WCE images, specifically focusing on their efficacy in detecting and identifying gastrointestinal issues, particularly polyps, as elucidated within the existing body of literature.

Polypoid lesion detection methods involve identifying frames that contain polypoid lesions (these frames may encompass more than one lesion without distinguishing between single or multiple ones in a given image), and segmentation, which entails segmenting the mucosal area within a frame that contains lesion(s).

In the realm of image classification, deep learning has achieved significant success due to its capacity to autonomously acquire potent feature representations from data. Despite its widespread application in natural images, the deployment of deep learning in the diagnosis of WCE images has been comparatively limited.

In the domain of polyp segmentation, recent strides have been made, exemplified by the Feedback Attention Network (FANet). Extending the advancements in polyp segmentation, Tomar et al. introduce a groundbreaking approach termed FANet [8]. This innovative architecture leverages the insights gained from each training epoch to refine subsequent predictions. FANet unifies the prior epoch’s mask with the feature map of the current training epoch, implementing hard attention at various convolutional layers. Notably, the model enables iterative rectification of predictions during test time. Tomar et al. demonstrate the efficacy of FANet through substantial improvements in segmentation metrics across seven publicly available biomedical imaging datasets. The integration of feedback mechanisms in FANet showcases its potential to enhance the precision and robustness of biomedical image segmentation. Polyp segmentation poses significant challenges due to the diversity in size, colour, and texture, compounded by indistinct boundaries between polyps and mucosa. A notable solution in recent literature is the Parallel Reverse Attention Network (PraNet), proposed by Fan et al. [9]. PraNet employs a Parallel Partial Decoder (PPD) to aggregate features, generating a global map for guidance. The Reverse Attention (RA) module mines boundary cues, fostering recurrent cooperation between areas and boundaries, thereby enhancing segmentation accuracy. The efficacy of PraNet, positions it as a promising approach in advancing precise polyp segmentation in colonoscopy images. Additionally, the model integrates a parallel-partial decoder to boost its performance, a concept that has been implemented in various innovative architectures such as AMNet [10]. AMNet builds upon the edge-detection capabilities originally employed in PraNet, further improving the segmentation process.

Another noteworthy attention-based approach is illustrated in GMSRF-Net. In this model, multiple-resolution scales are uniformly fused throughout the architecture, presenting a novel method for attention. Multi-scale approaches have been explored in the context of polyp segmentation, including MSNet [11]. While the initial success of MSNet was noteworthy, recent progress has significantly refined its performance. For polyp segmentation, ensemble methods have gained traction in recent years, leading to the evolution of dual-encoder and/or dual-decoder architectures, as evidenced by Galdran et al. [12]. The study highlights the promising outcomes achieved with a dual encoder-decoder approach. However, it is crucial to note that the implementation followed a sequential structure for the dual model, where the output of one encoder-decoder acted as the input for the subsequent one. Additionally, the reliance on existing pretrained architectures limited the introduction of novel components in the network design [12].

Yang et al. developed a colon polyp detection and segmentation algorithm based on an improved MRCNN, involving training large-scale datasets, extracting the initial model, and retraining it with smaller private datasets from patients [13]. In the pursuit of lightweight network structures with high classification accuracy, Wang et al. combined VGGNets and ResNets models with global average pooling, leading to the development of two new models, VGGNets gap and ResNets gap, featuring reduced parameters without compromising performance [14]. Manouchehri et al. presented a novel convolutional neural network designed for polyp frame detection, based on the VGG network. Moreover, they introduced a comprehensive convolutional network coupled with an efficient post-processing algorithm for polyp segmentation [15].

In the pursuit of refining polyp detection effectiveness, several algorithms rooted in the YOLO series were created. Guo et al. introduced an automated polyp detection algorithm that incorporates the YOLO-V3 structure along with active learning [16]. Similarly, Cao et al. contribute a novel approach to gastric polyp detection by introducing a feature extraction and fusion module integrated with the YOLOv3 network [17]. Their methodology surpasses alternative approaches, particularly excelling in the detection of small polyps. This effectiveness is attributed to the module’s capacity to seamlessly combine semantic information from high-level feature maps with low-level feature maps, thereby enhancing the detection capabilities for smaller polyps. This study underscores the significance of feature extraction and fusion in optimising deep neural networks for improved gastric polyp detection. Nogueira-Rodriguez et al. introduced a deep learning model designed for automatic polyp detection based on YOLO-V3, including an object tracking step to mitigate false positives. Model performance was augmented through training with a specialized dataset featuring a substantial number of polyp images [18]. Hoang et al. introduced a capsule endoscope system designed for small bowel and colon applications, featuring 5D position sensing and real-time automatic polyp detection. This system utilizes a YOLO-V3-based algorithm for the detection of real-time polyps, achieving an average precision of 85% [19].

In the contribution to real-time polyp detection, Pacal and Karaboga elevate the YOLOv4 algorithm by integrating Cross Stage Partial Networks (CSPNet), switching from Mish to Leaky ReLu activation, and adopting Complete Intersection over Union (CIoU) loss over Distance Intersection over Union (DIoU) [20]. Beyond algorithmic modifications, diverse architectural structures like ResNet, VGG, DarkNet53, and Transformers enhance YOLOv3 and YOLOv4 performance. Augmenting the method’s effectiveness, the study employs various data augmentation techniques, an ensemble learning model, and NVIDIA TensorRT for post-processing. To ensure objective comparisons, only public datasets are utilised, aligning with the MICCAI Sub-Challenge on Automatic Polyp Detection in Colonoscopy. This study presents a comprehensive approach, yielding substantial improvements in real-time polyp detection. In a related context, Lee et al., contribute to colon polyp detection using YOLOv4, emphasising its multiscale learning capability [21]. They enhance this feature by introducing additional scales and optimising the activation function, leading to continuous feature extraction during layer weight updates. The comprehensive approach results in a significant performance boost, achieving a mean Average Precision (mAP) of 98.36. This study highlights effective strategies for refining network structure and employing data augmentation to improve colon polyp detection.

Wan and colleagues proposed a YOLO-V5-based model designed for real-time polyp detection [22]. Their approach integrates a self-attention mechanism, enhancing relevant features while diminishing less pertinent ones, ultimately leading to improved performance. In a comprehensive experimental investigation, Pacal et al. examined innovative datasets, SUN and PICCOLO, employing the Scaled YOLO-V4 algorithm [23]. The results showcased remarkable success in polyp detection for both the SUN and PICCOLO datasets, positioning the Scaled YOLO-V4 algorithm as one of the most suitable object detection methods for large-scale datasets. Souaidi et al. introduced a hybrid method based on SSDNet for polyp detection, focusing on modelling the visual appearance of small polyp areas [24]. Notably, this method incorporated modified initial-A modules instead of simple convolution layers to enhance intermediate feature maps. Experimental validation of the proposed method’s polyp detection performance was conducted using public datasets. In the work by Karaman and Pacal [25], they enhance YOLO-based object detection through the utilisation of the ABC algorithm. This optimisation results in a 3% enhancement in real-time polyp detection using YOLO-V5 on the SUN and PICCOLO datasets. The research presents an adaptable and personalised methodology that is applicable to diverse datasets and any YOLO-based algorithm [26].

In reviewing the existing studies on the analysis of WCE images, it becomes evident that while numerous methodologies exhibit progress, certain gaps and limitations persist in the current body of literature. Some models, though initially showing promise, require further refinement and validation across diverse datasets to establish their generalizability. Moreover, the overall assessment indicates a need for more comprehensive investigations into real-time capabilities, dataset adaptability, and potential limitations in various clinical scenarios. These research gaps present opportunities for future studies to address and enhance the effectiveness of methodologies for detecting and identifying GI issues in WCE images.

Table 1 presents a summary of the aforementioned prevailing methodologies for the analysis of WCE images, specifically focusing on their efficacy in detecting and identifying GI issues.

## 3. Material and Applied Method

### 3.1. Network Configuration

The models were created using the PyTorch version 2.1.0, with all training and inference processes running on the Google Colab cloud server, equipped with an NVIDIA T4 GPU (16 GB RAM), an Intel Xeon CPU at 2.20 GHz, 12 GB of RAM, and a high-speed SSD.

### 3.2. Dataset

We have used KID Dataset which is an accessible repository aimed at catalysing the development and assessment of innovative MDSS solutions [27,28,29]. Designated as KID Dataset 2, this compilation comprises meticulously annotated WCE images, captured throughout the GI tract using a MiroCam capsule endoscope with a resolution of 360 × 360 pixels. The dataset features a comprehensive array of anomalies, categorised into 303 images of vascular anomalies (small bowel angiectasias, lymphangiectasias, and luminal blood), 44 images of polypoid anomalies (lymphoid nodular hyperplasia, lymphoma, and Peutz-Jeghers polyps), 227 images of inflammatory anomalies (ulcers, aphthae, mucosal breaks with surrounding erythema, cobblestone mucosa, luminal stenoses and/or fibrotic strictures, and mucosal/villous edema), and 1778 normal images derived from the oesophagus, stomach, small bowel, and colon. The strength of the dataset lies not only in its breadth but also in the collaborative efforts of the KID working group, consisting of six centres that have contributed anonymised, annotated Capsule Endoscopy (CE) images and videos from various CE models. This collaborative endeavour has resulted in a comprehensive repository exceeding 2500 annotated CE images and 47 videos, covering diverse categories such as normal CE, vascular lesions, inflammatory lesions, lymphangiectasias, and polypoid lesions [28]. To ensure standardised representation, lesion categorisation adheres to the CE Structured Terminology (CEST), providing a robust foundation for the utility of the dataset [30]. The dataset maintains a commitment to high-quality original resolution, avoiding distortions introduced by additional compression. Image formats align with ISO/IEC 15948 [31] PNG standards, a platform-independent, lossless compression format, with alternative acceptable standards including ISO/IEC 14496-12 [32], MPEG-4, AVC (Advanced Video Coding), and H.264 for videos, encompassing F4V and FLV (Flash video) formats. To facilitate the training and evaluation of models, we performed a split of the dataset into training (70%), validation (15%), and test (15%) sets. This split ensures that our models are trained on a diverse range of data, validated for generalisation, and tested on unseen samples. The split was conducted randomly while maintaining the distribution of anomaly and normal images across the sets. Facilitating the annotation process is the integration of software tools for video manipulation and image annotation on the KID website. Annotations are supported by an open-access, platform-independent tool called Ratsnake, enabling both semantic and graphic annotations [33]. Semantic annotation utilises textual labels and adheres to standard web ontology language description logics (OWL DL) [34].

### 3.3. Framework and Methodology

Our main focus in enhancing our segmentation strategy revolves around amplifying segmentation capabilities, specifically targeting polypoid lesions in WCE images. To attain this objective, we harness the power of YOLO-V8 [35], an advanced iteration of the original YOLO [36].

#### 3.3.1. YOLO-V8 Architecture

YOLO-V8 has attained cutting-edge performance by refining the model structure, incorporating both anchor box and anchor-free schemes, and integrating a diverse array of data augmentation techniques. Our deep learning framework is structured around five distinct-sized versions of YOLO-V8, labelled as n, s, m, l, and x, each characterised by varying channel depth and filter numbers as outlined in Table 2 [35]. In the backbone architecture, all five models are employed, capitalising on their balanced blend of segmentation accuracy and processing speed. Integrating YOLO-V8 into our computer vision project brings forth several advantages, with heightened accuracy emerging as a primary asset compared to its predecessors within the YOLO model lineage. YOLO-V8 extends its support to a spectrum of tasks, encompassing object segmentation, instance segmentation, and image classification, thereby enhancing its versatility for diverse applications. Positioned as the latest evolution in YOLO’s object segmentation paradigm, YOLO-V8 places a central emphasis on augmenting both accuracy and efficiency relative to its forerunners. This version brings comprehensive improvements, such as an advanced network design, a new take on anchor boxes, and an updated loss function, all contributing to significantly better segmentation accuracy.

The superiority of YOLO-V8 shines through its exceptional accuracy, making it a competitive choice among cutting-edge object segmentation frameworks. Engineered with efficiency at its core, YOLO-V8 is tailored for seamless execution on standard hardware, making it a practical and feasible choice for various object segmentation tasks, including those involving edge computing scenarios. The incorporation of anchor boxes in YOLO-V8 serves to align predicted bounding boxes with ground-truth bounding boxes, thereby further enhancing the overall accuracy of the object segmentation process.

The training process of YOLO-V8 is set to achieve significantly faster speed when compared to two-stage object segmentation models, presenting it as an efficient option for projects with stringent time constraints. Diverging from the structure seen in ultralytics /YOLO-V5 [37], substantial modifications were applied to the system’s backbone. This involved the replacement of C3 with C2f and the integration of the ELAN principle from YOLO-V7 [38]. Notably, the initial 6 × 6 convolution in the stem was replaced with a 3 × 3 convolution, augmenting the model’s capability to capture more comprehensive gradient flow information.

The C3 module consists of three ConvModules and n DarknetBottleNecks, while the C2f module is made up of two ConvModules and n DarknetBottleNecks, which are interconnected via Split and Concat operations. The ConvModule follows a Conv-BN-SiLU activation sequence, with ’n’ representing the number of bottleneck units. In the C2f module, the outputs from the Bottleneck, which includes two 3 × 3 convolutional layers with residual connections, are merged [1].

**Figure 2:**
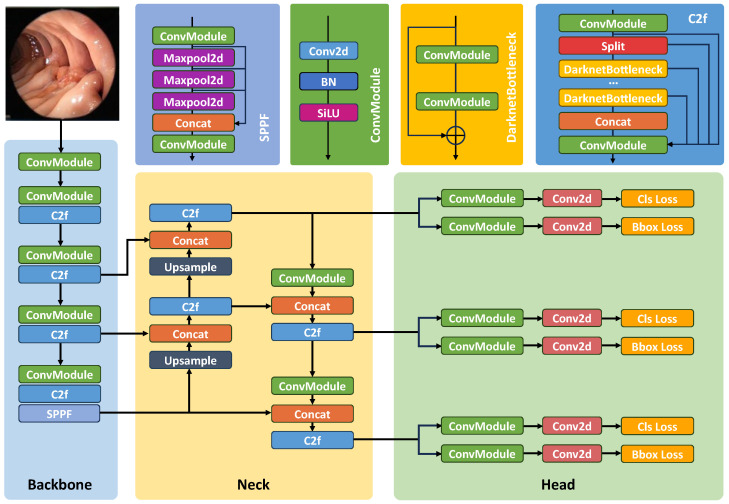
Architecture of YOLO-V8 with backbone and head components.

Deviating from the YOLO-V5 architecture, which incorporates a linked head, this methodology introduces a separated head, distinctly isolating the classification and segmentation elements. Significantly, the model eliminates the objectness branch, maintaining only the classification and regression branches. Anchor-Base utilises numerous anchors within the image to calculate the four offsets representing the regression object’s location in relation to the anchors. This mechanism enhances the accurate determination of the object’s location by employing corresponding anchors and offsets. The architectural representation of the model can be observed in Figure 2.

#### 3.3.2. Training the Model

In the training phase of the model, the Task Aligned Assigner from Task-aligned One-stage Object Detection (TOOD) [39] has been employed to assign positive and negative samples. This method selects positive samples through an assessment that combines the weighted scores from both classification and regression, as detailed in Equation (1).

(1) $$t=s^{\alpha }\cdot u^{\beta }$$

In this context, *s* represents the predicted score associated with the identified class, and *u* represents the Intersection over Union (IoU) between the predicted and the actual bounding box. Moreover, the model incorporates branches for classification and regression. The classification branch employs the Binary Cross-Entropy (BCE) Loss, as depicted by the subsequent equation:

(2) $${\mathrm{Loss}}_{n}=-w\left[y_{n}\log\left(x_{n}\right)+\left(1-y_{n}\right)\log\left(1-x_{n}\right)\right]$$

where *w* represents the weight, $y_{n}$ represents the labelled value, and $x_{n}$ is the predicted value generated by the model.

Regarding the regression branch, Distribute Focal Loss (DFL) [40] and Complete Intersection over Union (CIoU) Loss [41] have been utilised. DFL is employed to widen the probability distribution around the object *y*, and its equation is expressed as follows:

(3) $$\mathrm{DFL}\left(S_{n},S_{n+1}\right)=-\left(\left(y_{n+1}-y\right)\log\left(S_{n}\right)+\left(y-y_{n}\right)\log\left(S_{n+1}\right)\right)$$

Here, the equations for $S_{n}$ and $S_{n+1}$ are presented below:

(4) $$S_{n}=\left(\frac{y_{n+1}-y}{y_{n+1}-y_{n}}\right),\;S_{n+1}=\left(\frac{y-y_{n}}{y_{n+1}-y_{n}}\right)$$

The $CIoU_{Loss}$ introduces an influential factor into the DistanceIoU(DIoU) Loss [42], considering the aspect ratio of both the prediction and the ground truth bounding box. The equation is as follows:

(5) $$CIoU_{Loss}=1-IoU+\frac{{Distance}_{2}^{2}}{{Distance}_{C}^{2}}+\frac{v^{2}}{\left(1-IoU+v\right)}$$

where, *v* stands for the parameter that measures the consistency of the aspect ratio [42].

**Figure 3:**
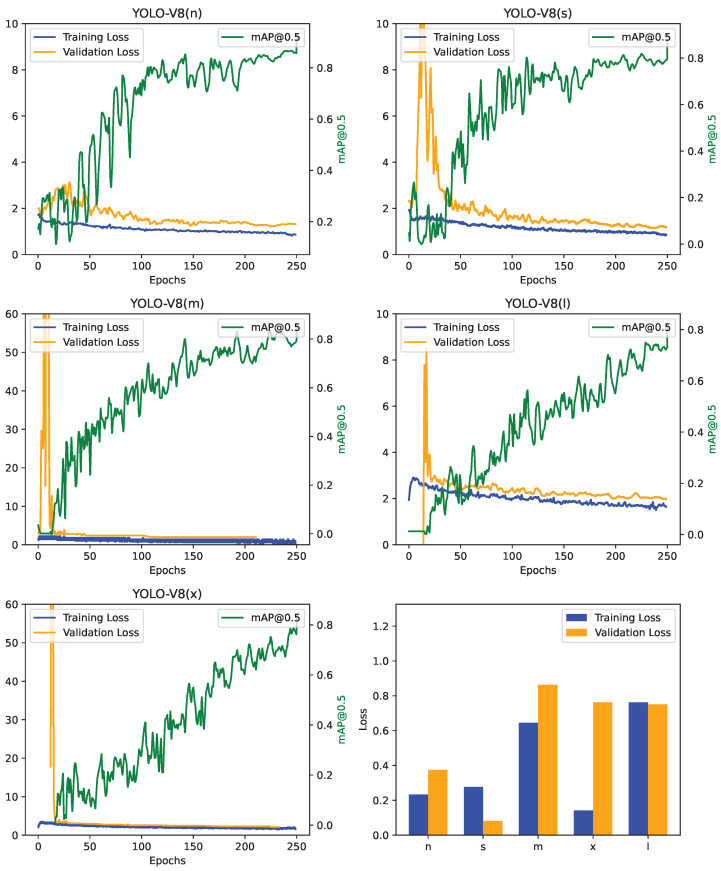
Training and Validation results diagrams of applied methods in 5 versions of YOLO-V8.

Figure 3 illustrates the training results diagrams for the applied method, encompassing five iterations of distinct versions of YOLO-V8. These plots, designated as ‘n’, ‘s’, ‘m’, ‘x’, and ‘l’, represent distinct versions or settings of the YOLO model. Starting with YOLO-V8(n), both training and validation losses exhibit a consistent decrease over time, indicative of effective learning and good generalisation, as the validation loss closely mirrors the training loss. YOLO-V8(s) follows a similar trend, with initial volatility settling into convergence. YOLO-V8(m) exhibits initial extreme volatility, with a sudden spike in loss followed by stabilisation, potentially signalling an issue with early epochs. YOLO-V8(l) and YOLO-V8(x) share a pattern of initial spikes, yet stabilise, with their training and validation losses converging. The bottom right bar plot provides a summarised view of average or final loss values for each model version, revealing ‘n’ and ‘l’ as top performers and ‘x’ with the highest loss. Analysing initial spikes, convergence patterns, and potential overfitting, it is apparent that despite the initial challenges, all models eventually converge without signs of overfitting. Choosing the optimal model involves considering both final loss values and loss curves, prioritising stability and lower validation loss for robust generalisation to unseen data.

## 4. Results and Discussion

### 4.1. Evaluation Metrics

This paper evaluates the performance of the algorithm in segmenting polypoid lesions using three key metrics: Precision, Recall, and Dice score. The equations for these metrics are provided below:

(6) $$Precision=\frac{TP}{TP+FP}$$

(7) $$Recall=\frac{TP}{TP+FN}$$

(8) $$Dice\;score=\frac{2\times TP}{2\times TP+FP+FN}$$

Within these indicators, TP (true positives) refers to the number of points correctly identified as part of polypoid lesions, indicating precise detection and labelling. FN (false negatives) denotes the number of points where polypoid lesions were present but not accurately detected, highlighting missed lesion areas. TN (true negatives) encompasses points correctly identified as not being part of polypoid lesions, contributing to the model’s specificity assessment. Finally, FP (false positives) represents points incorrectly classified as part of polypoid lesions, indicating areas mistakenly identified as lesions. Together, these metrics offer a comprehensive assessment of the model’s performance in distinguishing between polypoid and non-polypoid lesions, crucial for evaluating its overall effectiveness and reliability.

Precision evaluates the proportion of accurately labelled polypoid lesions among all the predicted occurrences of such lesions. It functions as a metric that gauges the precision of predictions. In polypoid lesion segmentation, precision signifies the level of confidence in correctly identifying a positive segmentation. A heightened precision value proves beneficial by minimising the occurrence of false alarms, subsequently alleviating financial and psychological stress for patients. Precision(B) (Bounding Box Precision) specifically assesses precision at an IoU (Intersection over Union) threshold of 0.5 for bounding boxes. It measures how accurately the model identifies polypoid lesions within bounding boxes, considering cases where there is at least 50% overlap with the ground truth bounding boxes.Precision(M) (Multiple IoU Precision) evaluates precision performance across a range of IoU thresholds, typically 0.5, 0.75, etc. This provides a thorough evaluation of the model’s accuracy in identifying objects that have different levels of spatial intersection with the actual data, providing an understanding of its consistency in various situations.

Conversely, Recall signifies the portion of detected objects. In the context of polypoid lesion segmentation, this metric holds substantial importance, as a higher recall guarantees that a greater number of patients undergo timely follow-up examinations and receive suitable treatment. As a result, this could lead to diminished mortality rates and prevent unwarranted costs for patients. Recall quantifies the ratio of identified polypoid lesions from all images containing such lesions. Recall (B) typically stands for Recall at IoU (Intersection over Union) threshold of 0.5 for bounding boxes. It measures how well the model is able to recall (detect) objects with bounding boxes that have at least 50% overlap with the ground truth bounding boxes. Recall (M) often stands for Recall at multiple IoU thresholds, usually 0.5, 0.75, etc. It means evaluating recall performance across a range of IoU thresholds, providing a more comprehensive assessment of the model’s ability to detect objects.

mAP50 (B) stands for mean Average Precision at 50% IoU threshold for bounding boxes. Average Precision (AP) is a metric that considers both precision and recall at different confidence thresholds. mAP50 (B) specifically looks at the average precision when considering bounding boxes with at least 50% overlap with the ground truth. mAP (M) stands for mean Average Precision at multiple IoU thresholds. It is a broader evaluation that looks at the average precision across a range of IoU thresholds, providing a more complete picture of the model’s performance. Notably, mAP50-95 (B) stands out as a significant metric, capturing the model’s performance across a broader range of detection thresholds, akin to the mAP50 measure.

Dice score measures the overlap between predicted and ground truth masks, offering a concise and intuitive measure of segmentation accuracy in the context of polypoid lesions. It ranges from 0 to 1, with 1 indicating a perfect match, making it valuable for assessing the precision of delineating regions of interest in medical image analysis.

### 4.2. Performance of the Applied Method

Table 3 elaborated on the hyperparameter setting by providing the key metrics of each model’s performance on the validation dataset. The results presented in Table 3 underscore the outstanding performance achieved by various versions of YOLO-V8 in tasks associated with polypoid lesion segmentation.

As a concluding step, we assessed the efficacy of our approach on the KID dataset, contrasting its outcomes with those obtained from other state-of-the-art methodologies. We utilised the generated dataset to evaluate different state-of-the-art models. Table 4 shows the evaluation results of different algorithms. This table presents a comprehensive comparison of various detection methods, evaluating their performance in terms of precision, recall, and dice score. Notably, the Resnet family exhibits high performance. This suggests their efficiency in achieving a balance between model complexity and performance. NanoNet-A, NanoNet-B, and NanoNet-C demonstrate competitive results, particularly NanoNet-B, which achieves impressive recall with a significantly lower number of parameters, making it a compelling choice for resource-efficient applications.

**Figure 4:**
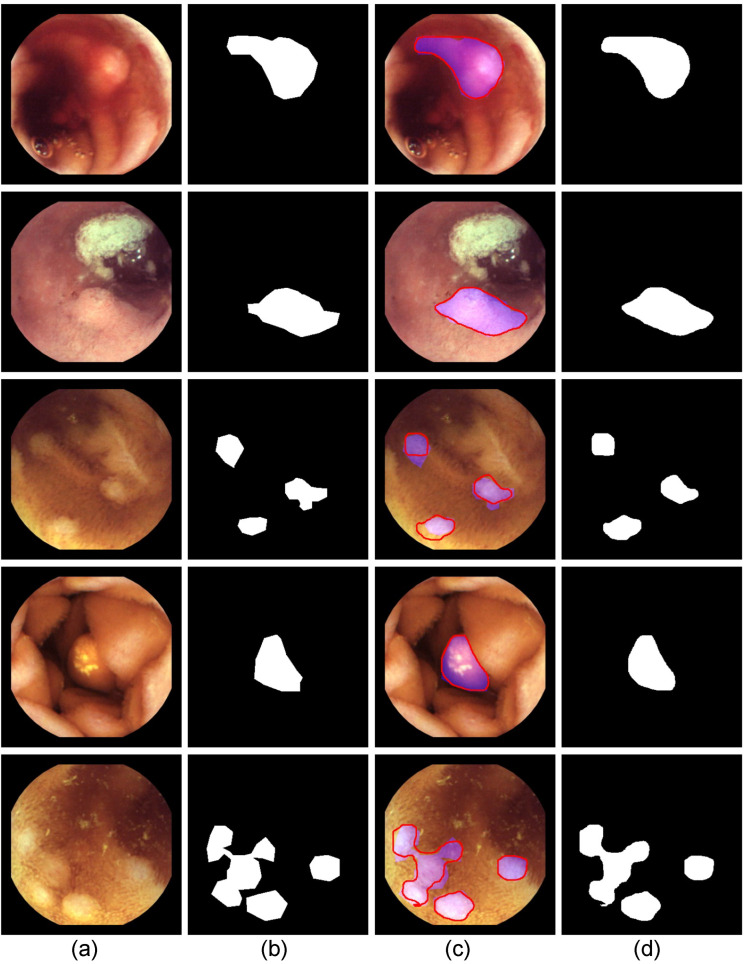
Segmentation results from YOLO-V8 m showcase subsets for single and multi-polypoid lesion instances in our dataset’s test images: (**a**) Original Images; (**b**) Ground Truth Binary Mask; (**c**) Segmented polypoid lesion(s) outlined in red contour with the ground truth depicted in purple; and (**d**) Segmented polypoid lesion(s) Binary Mask.

Visual outcomes of the applied method, along with the detected polypoid lesions and their corresponding outlines, are illustrated in Figure 4.

### 4.3. Discussion

Incorporating YOLO-V8 into computer vision projects provides heightened accuracy compared to preceding YOLO models, rendering it a versatile solution for tasks such as object segmentation, instance segmentation, and image classification. This latest iteration emphasises the enhancement of accuracy and efficiency through optimised network architecture, a revamped anchor box implementation, and a modified loss function. YOLO-V8 showcases superior accuracy, establishing itself as a robust contender among state-of-the-art object detection models. Engineered for efficiency, it operates seamlessly on standard hardware, rendering it a practical choice for real-time object segmentation tasks.

This study underscores the critical importance of early detection and prevention in the context of GI tract disorders, particularly SBTs and CRC, and introduces and evaluates the YOLO-V8 deep learning model for automated polypoid lesion segmentation, addressing the pressing need for efficient and accurate computer-aided diagnosis.

The YOLO-V8 method exhibits diverse performance across its iterations (n, s, m, l, x), with YOLO-V8 m notably standing out due to its exceptional precision (98%) and recall (97.9%), making it a promising choice for accurate polypoid lesion segmentation, as indicated in Table 3. Comparatively, our study using YOLO-V8 m surpasses the performance of various state-of-the-art segmentation algorithms, as illustrated in Table 4. YOLO-V8 m outperforms other architectures in terms of precision, recall, and dice score, showcasing its effectiveness in lesion detection. When considering application-specific requirements, Resnets are recognised for their efficiency with fewer parameters, while NanoNet-B excels in resource efficiency without compromising performance. Despite YOLO-V8 m’s impressive metrics, it comes with a larger model size (27 M parameters). This emphasises the need to carefully balance model complexity and performance when selecting a segmentation method tailored to a particular use case, highlighting the comprehensive insights.

The YOLO-V8 architecture has been intricately designed to facilitate optimal information flow, adeptly capturing intricate features relevant to the segmentation of polypoid lesions while minimising unnecessary computational burden. This carefully crafted design ensures smooth processing without compromising accuracy, placing a strong emphasis on attending to various scales and resolutions present in the input image. YOLO-V8 effectively addresses the multiscale nature inherent in polypoid lesions, allowing for detailed discernment at different levels and significantly contributing to heightened precision and recall compared to larger, less adaptable models. Moreover, the study’s reliance on the KID Dataset, validated by the KID working group, not only enhances the credibility of the findings but also underscores the versatility of YOLO-V8 in adapting to a wide array of datasets.

By incorporating advanced training strategies and data augmentation techniques, YOLO-V8 is adeptly designed to learn from diverse datasets, a critical feature for polypoid lesion detection where size, shape, and appearance vary widely. Its adaptability significantly boosts the model’s generalisation capabilities and robustness, leading to enhanced performance on previously unseen data. YOLO-V8 marks a significant evolution from its predecessor, YOLO-V5, through crucial updates and modifications that sharpen its accuracy. These advancements enable YOLO-V8 to outperform older, more resource-heavy models, showcasing its superiority in the field.

### 4.4. Limitations and Future Scopes

The approach presented signifies a substantial leap beyond its predecessors in the realm of YOLO algorithms, showcasing notable enhancements in hyperparameter optimisation while addressing both temporal and financial constraints. However, the study’s broader impact was limited by the lack of a comprehensive public dataset. Despite achieving positive outcomes, specific datasets from existing literature were omitted because of the limited availability of polyp images and a constrained patient pool, underscoring the essential dependence on robust datasets for deep learning approaches and the imperative need for diverse and expansive datasets to showcase optimal performance. Nevertheless, the methodology has made substantial progress in improving the speed and efficiency of real-time detection, outperforming existing methods and previous iterations of YOLO. Efforts are ongoing to leverage these advancements in clinical applications by integrating available datasets. Nonetheless, an acknowledgement exists regarding the importance of datasets encompassing a more extensive range of WCE images, reflecting varied patient demographics and geographical origins [48]. In our forthcoming research, we aim to broaden our investigative scope to facilitate more comprehensive statistical evaluations. Our strategy includes the implementation of sophisticated statistical tests to delve into the relationships between model characteristics and performance metrics, with a specific emphasis on comparing models such as YOLO-V8 using advanced methodologies. This approach seeks to furnish a more profound and accurate assessment of their effectiveness.

## 5. Conclusions

In this study, we addressed the pressing issue of identification of polypoid lesions in WCE images, a critical aspect of preventing cancer. We introduced a detection system utilising YOLO-V8 models to aid gastroenterologists in identifying and categorising polypoid lesions accurately and efficiently. We highlighted the significance of timely polypoid lesion segmentation and removal in reducing cancer-related mortality. To this end, we harnessed the potential of WCE technology, which offers an innovative approach to diagnosing GI conditions without resorting to invasive procedures.

The KID dataset, a comprehensive repository of video capsule endoscopy images, served as the foundation for our research. We curated this dataset to focus on the polypoid lesion class, generating corresponding ground truth masks for precise segmentation tasks.

The YOLO-V8 model emerged as our chosen deep learning architecture due to its state-of-the-art accuracy, versatility, and efficiency. Our meticulous data augmentation techniques ensured the diversity and robustness of the training dataset, further enhancing the model’s performance. Upon evaluating the applied approach, we achieved impressive results, demonstrating the capability of our model in polypoid lesion segmentation. The metrics of precision, recall, and mean average precision were utilised for the assessment of the different types of applied models. Among the variants and other models, YOLO-V8 m stands out with a precision of 98%, recall of 97.9% and also strikes a balance between accuracy and computational efficiency, making it a promising choice.

This research has the potential to serve as a foundational reference for future studies on polypoid lesion segmentation and classification. Given the rapid advancements in the field of computer vision in recent years, the creation of a benchmark dataset becomes crucial. Our aspiration is that the dataset generated in this study will significantly accelerate the progress of computer-aided cancer diagnosis.

## Figures and Tables

**Table 1:** Recent related studies.

Study	Year	Method
Song et al. [10]	2022	AMNet
Tomar et al. [8]	2022	FANet
Zhao et al. [11]	2021	MSNet
Galdran et al. [12]	2021	Double Encoder-Decoder Network
Yang et al. [13]	2020	MRCNN
Wang et al. [14]	2020	VGGNet + ResNet
Haj-Manouchehri et al. [15]	2020	CNN + VGGNet
Guo et al. [16]	2022	YOLO-V3
Cao et al. [17]	2021	YOLO-V3
Nogueira et al. [18]	2022	YOLO-V3
Hoang et al. [19]	2021	YOLO-V3
Pacal et al. [20]	2021	YOLO-V4
Lee et al. [21]	2022	YOLO-V4
Wan et al. [22]	2021	YOLO-V5
Pacal et al. [23]	2022	Scaled YOLO-V4
Souaidi et al. [24]	2022	SSDNet
Karaman et al. [25]	2023	ABC algorithm with YOLO-based
Karaman et al. [26]	2023	YOLO Algorithms

**Table 2:** Model specifications.

Model	d (depth_multiple)	w (width_multiple)	r (Ratio)
YOLO-V8 n	0.33	0.25	2.0
YOLO-V8 s	0.33	0.50	2.0
YOLO-V8 m	0.67	0.75	1.5
YOLO-V8 l	1.00	1.00	1.0
YOLO-V8 x	1.00	1.25	1.0

**Table 3:** Training and deploying the five iterations of YOLO-V8: Evaluating performance and outcomes.

YOLO Type	Precision (B)	Recall (B)	mAP50 (B)	mAP50-95 (B)	Precision (M)	Recall (M)	mAP50 (M)	mAP50-95 (M)	Dice score	Parameters (M)	Image Segmentation Time on CPU (s)	Image Segmentation Time on T4 GPU (s)
YOLO-V8 n	98.8	81.8	93.1	73.6	98.8	81.8	93.1	70.7	89.57	3	0.3322	0.0284
YOLO-V8 s	96.3	91.8	90.3	91.8	96.3	91.8	91.05	88.8	93.99	11	1.0768	0.0388
YOLO-V8 m	98	97.9	86.04	86.3	98	97.9	86.03	83.5	97.94	27	2.0339	0.055
YOLO-V8 l	92.4	93.6	82.7	86.07	80.3	80.6	80.9	83.7	92.99	45	3.8540	0.0652
YOLO-V8 x	85.2	80.6	84.4	86.6	85.2	80.6	83.9	85.6	82.78	71	5.8717	0.1045

**Table 4:** Comparative analysis of different algorithms: performance results.

Study	Dataset	Architecture	Precision	Recall	Dice Score
Delagah et al. [43]	Kvasir-Capsule	SVM	99.5%	97.5%	98.47
Amiri et al. [44]	Kvasir-Capsule	Fast ROI	94.3%	90.9%	92.56
Sornapudi et al. [45]	Mayo Clinic	Resnet-101(Pre-TrainedWeights:COCO)	94.03%	94.03%	94.03
Sornapudi et al. [45]	Mayo Clinic	Resnet-101(Pre-TrainedWeights:ImageNet)	82.05%	95.52%	87.40
Sornapudi et al. [45]	Mayo Clinic	Resnet-101(Pre-TrainedWeights:Balloon)	98.46%	95.52%	96.96
Sornapudi et al. [45]	Mayo Clinic	Resnet-50(Pre-TrainedWeights:COCO)	94.12%	95.52%	94.82
Sornapudi et al. [45]	Mayo Clinic	Resnet-50(Pre-TrainedWeights:COCO)	92.06%	86.57%	89.21
Sornapudi et al. [45]	Mayo Clinic	Resnet-50(Pre-TrainedWeights:Balloon)	90%	94.03%	91.96
Goel et al. [46]	AIIMS	DICR-CNN	90%	92%	91.43
Goel et al. [46]	KID	DICR-CNN	91%	95%	92.93
Souaidi et al. [24]	KID	Hyb-SSDNet	93.29%	89.4%	91.29
Jha et al. [47]	Kvasir-Capsule	NanoNet-A	93.2%	96.9%	94.04
Jha et al. [47]	Kvasir-Capsule	NanoNet-B	93%	98.8%	95.87
Jha et al. [47]	Kvasir-Capsule	NanoNet-C	92.3%	97.5%	94.85
Our Study	KID	YOLO-V8 m	98%	97.9%	97.94

## Data Availability

The KID dataset (https://mdss.uth.gr/datasets/endoscopy/kid/) accessed on 12 November 2023, used in this study is not publicly available. However, interested researchers can request access to the dataset by directly contacting the dataset providers.
